# NMDA receptor-dependent glutamate excitotoxicity in human embryonic stem cell-derived neurons

**DOI:** 10.1016/j.neulet.2013.03.010

**Published:** 2013-05-24

**Authors:** Kunal Gupta, Giles E. Hardingham, Siddharthan Chandran

**Affiliations:** a Cambridge Centre for Brain Repair, University of Cambridge, Cambridge CB2 0SZ, United Kingdom; b Centre for Integrative Physiology, University of Edinburgh, Edinburgh EH8 9XD, United Kingdom; c Centre for Neuroregeneration, University of Edinburgh, Edinburgh EH16 4SB, United Kingdom

**Keywords:** aCSF, artificial cerebrospinal fluid, AMPA, 2-amino-3-(5-methyl-3-oxo-1,2-oxazol-4-yl)propanoic acid, AMPAR, 2-amino-3-(5-methyl-3-oxo-1,2-oxazol-4-yl)propanoic acid receptor, D-APV, aminophosphonopentanoic acid, HESC, human embryonic stem cell, HPSC, human pluripotent stem cell, iPSC, induced pluripotent stem cell, NPC, neural precursor cell, NMDA, N-methyl-d-aspartic acid, NMDAR, N-methyl-d-aspartic acid receptor, Human pluripotent stem cells, MK 801, Excitotoxicity, Glutamate, Disease-modelling

## Abstract

Thanks to the development of efficient differentiation strategies, human pluripotent stem cells (HPSC) offer the opportunity for modelling neuronal injury and dysfunction in human neurons *in vitro*. Critically, the effective use of HPSC-derived neural cells in disease-modelling and potentially cell replacement therapies hinges on an understanding of the biology of these cells, specifically their development, subtype specification and responses to neurotoxic signalling mediators. Here, we generated neurons from human embryonic stem cells and characterised the development of vulnerability to glutamate excitotoxicity, a key contributor to neuronal injury in several acute and chronic neurodegenerative disorders. Over two months of differentiation we observed a gradual increase in responsiveness of neurons to glutamate-induced Ca^2+^ influx, attributable to NMDA receptor activity. This increase was concomitant with an increase in expression of mRNA encoding NMDA and AMPA receptor subunits. Differentiated neurons were vulnerable to glutamate excitotoxicity in a dose-dependent manner, which was reduced by NMDA receptor antagonists.

## Highlights

• Human embryonic stem cell derived neurons develop NMDA and AMPA receptors in culture.
• Enriched HESC-derived neurons develop functional glutamate responses *in vitro.*
• MK 801 confers a protective effect on glutamate-induced excitotoxic neuronal death.
• Studies using human pluripotent stem cells may aid the discovery of novel therapeutic targets.

## 1 Introduction

Glutamate excitotoxicity, both acute and chronic, has been implicated widely in the pathogenesis of a range of neurological diseases, including Alzheimer's disease, Huntington's disease and traumatic brain injury [25,28]. Knowledge of the role and mechanisms of glutamate excitotoxicity can potentially enhance our understanding of key downstream signalling processes that mediate neuronal death and identify novel therapeutic targets. A major cause of glutamate excitotoxicity is activation of the NMDA subtype of glutamate receptor (NMDAR), which mediates Ca^2+^-dependent cell death [26]. Most NMDARs contain two obligate GluN1 subunits plus two GluN2 subunits [11], of which there are four subtypes, GluN2A-D, with GluN2A and GluN2B predominant in the forebrain [30,40]. While physiological patterns of synaptic NMDAR activity have the potential to be neuroprotective [15,19,39], excessive or inappropriate activity is harmful. The work of many laboratories has advanced our understanding of how loss of glutamate homeostasis, ionic imbalance and inappropriate NMDAR activity can contribute to degenerative disorders [8,16,22,25,27].

Antagonism of pathological glutamate-signalling in rodent models of neurological diseases has yielded promising results [6,10], though unsuccessful clinical trials highlight the need for further studies [1,31]. Currently, excitotoxic neuronal cell death has primarily been characterised in rodent systems. Advances in human pluripotent stem cell (HPSC) technology permit the generation of enriched neurons of differing positional identities as well as astrocytes from human embryonic stem cells (HESC) and allow investigation of cell injury and rescue paradigms in a human *in vitro* system [12,13,23,24,35,38]. In light of the inter-species differences between rodent and human [34], studies utilising human *in vitro* systems may prove informative in interpreting the relevance of findings in rodent studies to the human pathological condition [3,13,18].

Here, we describe the functional maturation of HESC-derived neurons and the development of glutamate-responsiveness, in order to study neuronal responses to pathologically relevant glutamate concentrations [5].

## 2 Materials and methods

HESC Culture and neuralisation: The human embryonic stem cell (HESC) line H9 (Harvard University, Cambridge, MA) was cultured and passaged regularly on a feeder layer of irradiated mouse embryonic fibroblasts, and neuralised by established protocols [21]. HESC-derived neurons were generated by methodologies described by Koch et al. [23]. Enriched HESC-derived neural precursor cells (NPCs) were propagated in Advanced DMEM/F12, 1% Glutamax, 1% N2 supplement 1% penicillin-streptomycin, 0.1% B27 supplement, 10 ng/ml FGF and 10 ng/ml EGF. Coverslips were coated with Matrigel (BD Biosciences) for 1hr, diluted 1:30 in DMEM. NPCs were plated in NPC propagation medium without mitogens for 24 h and switched to Neurobasal-A, supplemented with 1% non-essential amino acids, 1% N2 supplement, 1% penicillin-streptomycin, 0.5% Glutamax, 10 ng/ml BDNF, 10 μM forskolin, 0.1 μM retinoic acid for neuronal differentiation. 50 μM DAPT [4] was included in the first medium change. These HESC-neuronal cultures are consistent with those described in Gupta et al. [13] in cellular composition; 95% were neurons and the remainder astrocytes, confirmed by TuJ1 and GFAP immunostaining. Differentiation duration was determined from the day of application of neuronal differentiation medium to NPCs. The day before experiments, HESC-derived neurons were removed from their trophic differentiation medium into a trophically deprived, glutamate-free “minimal medium” [13], which comprises 90% Salt-Glucose-Glycine (SGG) medium [2] and 10% MEM (Minimal Essential Medium, Invitrogen).

Calcium-imaging: The day before imaging, HESC-derived neurons were removed from their trophic differentiation medium into a trophically deprived, glutamate-free minimum medium as described above. HESC-neurons, identified by morphology and confirmed by TuJ1 immunostaining, were imaged at various time-points in artificial cerebrospinal fluid (aCSF) [39] on a 37 °C-heated stage. Cells were loaded with 11 μM Fluo-3 for 30 min and washed in aCSF. Coverslips were mounted in a perfusion chamber. Fluo-3 fluorescence images (excitation 488 nm/emission 520 nm) were taken at baseline and during saturating glutamate (200 μM) and NMDA (150 μM) stimulation. Glycine (100 μM) was applied as a co-agonist. D-APV (50 μM) was used as an NMDAR antagonist. For calibration, ionomycin was used to saturate the Fluo-3 signal (50 μM), followed by MnCl_2_ (10 mM) to quench. The quenched Fluo-3 signal corresponds to ~100 nM Ca^++^. The *K*_d_ of Fluo-3 was taken as 315 nM and free Ca^++^ concentration was calculated by the formula Ca^++^ = *K*_d_ (*F* − *F*_min_)/(*F*_max_ – *F*), where F is fluorescence. Images were taken every second for the duration of each experiment (~10 min) [17].

Gene expression analysis: Total RNA was isolated *via* the Absolutely RNA miniprep kit, as described [13]. cDNA synthesis performed *via* the AffinityScript cDNA (Stratagene) synthesis kit using oligo-dT and random hexamer primers, and qRT-PCR performed using Brilliant SYBR Green master mix (Stratagene), following the manufacturer's instructions. Forward and reverse primer sequences: *GRIN1* AGGAACCCCTCGGACAAGTT, CCGCACTCTCGTAGTTGTG; *GRIN2A* TGGACGTGAACGTGGTAGC, CCCCCATGAATGCCCAAGAT; *GRIN2B* TTCCGTAATGCTCAACATCATGG, TGCTGCGGATCTTGTTTACAAA; *GRIN2* *C* GAGTGGTCAAATTCTCCTACGAC, TGTAGTACACCTCCCCAATCAT; *GRIN2D* CTGGCCTCACTGGATCTGG, GGAAGGAAACCATAATCACGCA; *GRIA1* GGTCTGCCCTGAGAAATCCAG, CTCGCCCTTGTCGTACCAC; *GRIA2* AGTTTTCCACTTCGGAGTTCAG, CCAAATTGTCGATGTGGGGTG; *GRIA3* TCCGGGCGGTCTTCTTTTTAG, TGGGGAATCCTCCGTGAGAAT; *GRIA4* GCCATTGTCCCTGATGGAAAA, GAGGGGCAATAGCAATCTCTG; *GAPDH* AGGCTGGGGCTCATTTG, CAGTTGGTGGTGCAGGAG.

Electrophysiology: Whole-cell NMDA-evoked currents were recorded using an Axopatch 200B amplifier (Molecular Devices) using patch-pipettes made from thick-walled borosilicate glass with a tip resistance of 4–8 MΩ that were filled with an ‘internal’ solution that contained (in mM): K-gluconate 141, NaCl 2.5, HEPES 10, EGTA 11; pH 7.3 with KOH. Experiments were conducted at room temperature (18–21 °C) in an ‘external’ solution containing (in mM): NaCl 150, KCl 2.8, HEPES 10, CaCl_2_ 4, glucose 10, pH to 7.3 with NaOH. Picrotoxin (50 μM), strychnine (20 μM) and tetrodotoxin (300 nM) were also included. NMDAR-mediated currents were induced by the addition of saturating concentrations of NMDA (100 μM) and the co-agonist glycine (100 μM) to the external solution. Access resistances were monitored and recordings where this changed by >20% were discarded. Currents were filtered at 2 kHz and digitised online at 5 kHz *via* a BNC-2090A/PCI-6251 DAQ board interface (National Instruments, Austin, TX, USA).

Neuronal injury and Cell viability assays: The day before application of the excitotoxic insult, HESC-derived neurons were removed from their trophic differentiation medium into a trophically deprived, glutamate-free minimal medium. Neurons were treated with glutamate with and without MK801 (10 μM) [29]. 24 h after excitotoxic challenge, cell viability was quantified using CellTiter-Glo Luminescent Cell Viability Assay (Promega), following manufacturer's instructions. Neuronal injury experiments using high-dose NMDA (150 μM) [29], were performed in magnesium-free SGG medium [2]; cell viability was quantified after 24 h by the same protocol.

Data analysis: All experiments were performed with at least 3 biological repeats; within each, 3 technical repeats were generated. qPCR data were normalised to GAPDH. Data are presented as mean ± standard error of the mean; statistical analyses were performed in Microsoft Excel and GraphPad Prism 5 (GraphPad Software, Inc., San Diego) by univariate and two-way ANOVA with Newman-Keuls post-test and two-tailed Students’ *t*-test. *P* < 0.05 was considered statistically significant.

## 3 Results

### 3.1 Temporal profile of *in vitro* acquisition of HESC-derived neuronal glutamate responses

**Fig. 1:**
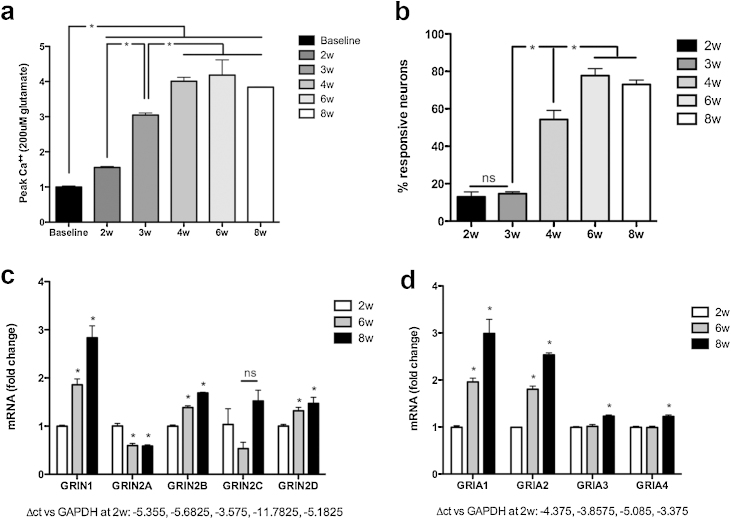
HESC-derived neurons were differentiated from 2 to 8-weeks and stimulated with 200 μM glutamate. (a) Development of glutamate-provoked calcium currents was dependent on culture duration. (b) The proportion of glutamate-sensitive neurons in the cultures also increased over time. Peak calcium current and maximal proportion of glutamate-responsive neurons were observed by 6-weeks in culture (*p < 0.001). HESC-derived neurons were investigated for NMDAR and AMPAR subunit expression at 2, 6 and 8-weeks in culture, by qRT-PCR. Significant increases in (c) NMDA and (d) AMPA subunit expression were observed (*p < 0.01). Comparison with baseline ct values at 2-weeks suggested that *GRIN1* and *GRIN2B*, and *GRIA1* and *GRIA2* were the most highly expressed receptor subunits at 6 and 8-weeks in culture.

In order to study excitotoxic responses in a human platform, enriched cultures of HESC-derived neurons were examined for functional glutamate-mediated responses. Neurons were stimulated with saturating glutamate (200 μM) and functional glutamate receptor activity was determined by calcium-imaging over increasing duration in culture. While initially unresponsive to glutamate (including doses of 1 mM glutamate: data not shown), HESC-derived neurons developed calcium currents in response to bath-extracellular glutamate application with increasing duration in culture. 2-week old HESC-derived neurons demonstrated no significant calcium influx, while later time points demonstrated significant calcium influx with glutamate treatment, with peak calcium currents increasing over time to maximal at 6 and 8-weeks duration in culture (*p* < 0.001, Fig. 1a). The proportion of HESC-derived neurons that exhibited glutamate-evoked calcium currents similarly showed a time-dependent increase from 13 ± 3% at 2-weeks and 15 ± 1% at 3-weeks, 54.4 ± 5% at 4-weeks, to 77.7 ± 4% and 73.0 ± 3% at 6 and 8-weeks respectively (*p* < 0.001, Fig. 1b). Thus, HESC-derived neurons acquired functional glutamate responsiveness over time in culture.

### 3.2 Development of functional glutamate responses coincides with expression of NMDA and AMPA receptor subunits

In order to determine if the temporal emergence of glutamate-responsiveness was due to increasing expression of glutamate receptors, we quantified the expression of NMDAR and AMPAR subunits in glutamate-unresponsive neurons at 2-weeks and in glutamate-responsive neurons at 6 and 8-weeks by qRT-PCR.

The NMDAR is a heterotetramer of two obligatory GluN1 subunits encoded by *GRIN1*, and two variable GluN2 subunits encoded by *GRIN2* genes. Expression of NMDAR subunit *GRIN1* increased from 2 to 6 and 8-weeks in culture by 1.9 ± 0.12-fold and 2.8 ± 0.25-fold respectively (*p* < 0.01). *GRIN2A* expression decreased at 6 and 8-weeks, to 0.6 ± 0.04-fold and 0.6 ± 0.03-fold respectively (*p* < 0.01). *GRIN2B* expression increased from 2-weeks to 1.4 ± 0.04-fold and 1.7 ± 0.01-fold and 6 and 8-weeks respectively (*p* < 0.05). There was no significant change in *GRIN2C* expression at 6 and 8-weeks (0.5 ± 0.13 and 1.5 ± 0.2-fold respectively). *GRIN2D* expression increased to 1.3 ± 0.07 and 1.5 ± 0.1-fold at 6 and 8-weeks respectively (*p* < 0.05, Fig. 1c). These data demonstrated an increase in expression of NMDAR subunits *GRIN1*, *GRIN2B* and *GRIN2D* in HESC-derived neurons over 8-weeks in culture, but not *GRIN2A*. Analysis of ct values suggested that NMDAR subunits 1 and 2B were the most highly expressed.

AMPAR subunit quantification showed that *GRIA1* expression increased significantly from 2 to 6 and 8-weeks, by 2.0 ± 0.08 and 3.0 ± 0.3-fold respectively (*p* < 0.01). *GRIA2* similarly increased with duration in culture, to 1.8 ± 0.07-fold and 2.5 ± 0.04-fold after 6 and 8-weeks respectively (*p* < 0.01). *GRIA3* and *GRIA4* showed no significant change in expression at 6-weeks (1.0 ± 0.04-fold and 1.0 ± 0.03-fold respectively) and a significant, although modest, increase in expression at 8-weeks (1.23 ± 0.027-fold and 1.22 ± 0.033-fold respectively, *p* < 0.05, Fig. 1d). These data demonstrated an increase in AMPAR expression of HESC-derived neurons over time in culture. Analysis of ct values suggested that AMPAR subunits 1 and 2 were the most highly expressed.

The NMDAR and AMPAR profiles were dynamic over the course of the period studied, with increasing expression of AMPAR and NMDAR subunits GluN1 and GluN2B coinciding with the development of functional glutamate responses *in vitro*. Given that during development rodent neurons undergo a transition from GluN2B subunit-containing NMDARs to GluN2A predominance [41], the NMDAR subunit profile in HESC-derived neurons may change further over time.

### 3.3 HESC-derived neurons express functional NMDA receptors

**Fig. 2:**
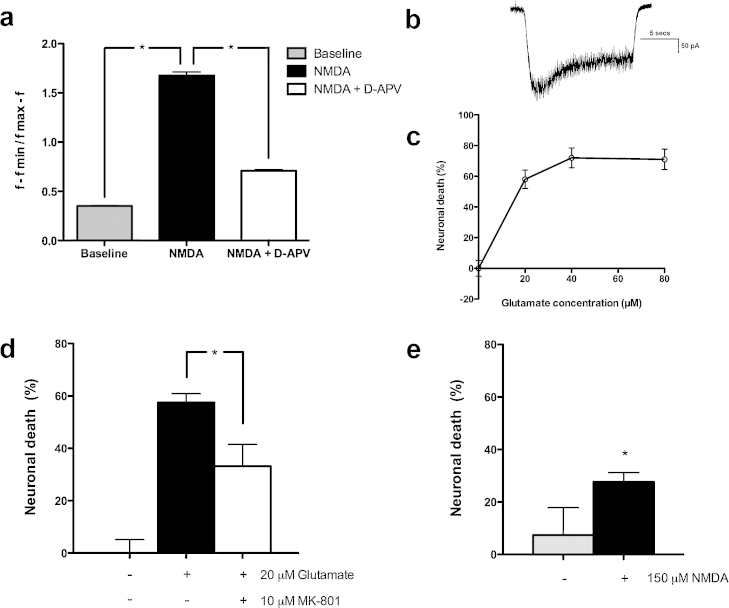
HESC-derived neurons were cultured for 6-weeks and stimulated with NMDA. (a) 100 μM NMDA elicited intracellular calcium currents, which were blocked by concurrent 50 μM D-APV administration (NMDAR antagonist) (*p < 0.05). (b) Neuronal responses to NMDA were confirmed by electrophysiology, demonstrating inward currents with 50 μM NMDA stimulation. (c) 7-week-old HESC-derived neurons were challenged with increasing glutamate concentrations in minimal medium, and cell viability assessed at 24 h after challenge. HESC-derived neurons were sensitive to glutamate-induced excitotoxic cell death, demonstrating 58 ± 6% cell death with 20 μM glutamate. (d) MK801, a non-specific NMDAR blocker, was added to HESC-derived neurons simultaneously with 20 μM glutamate challenge. Excitotoxic cell death was quantified after 24 h. MK801 co-administration significantly reduced glutamate-induced excitotoxic cell death (*p < 0.05). (e) HESC-derived neurons were challenged with 150 μM NMDA and neuronal death quantified after 24 h. Both conditions described were performed in magnesium-free SGG medium, and compared to trophically-deprived control medium (*p < 0.001).

In order to determine whether the glutamate-induced calcium influx was dependent on the expression of functional NMDARs, receptor-mediated calcium currents were quantified in 8-week HESC-derived neuronal cultures using NMDA-glycine co-treatment. Data demonstrated that HESC-derived neurons exhibited calcium currents upon NMDA application, which were blocked with application of D-APV, a selective NMDAR inhibitor (*p* < 0.05, Fig. 2a). NMDAR currents were further confirmed by patch-clamp electrophysiology (Fig. 2b).

### 3.4 HESC-derived neurons demonstrate excitotoxic cell death at pathophysiological glutamate concentrations, and are rescued by MK801-mediated NMDA receptor blockade

The susceptibility of 7-week-old HESC-derived neurons to glutamate-induced cell death was determined over a range of concentrations. With increasing glutamate concentrations, increasing cell death was observed: at 20 μM, 58% ± 5.98%; at 40 μM, 72 ± 6.45%; and at 80 μM, 71 ± 6.64% cell death was noted (Fig. 2c). 100% cell death was induced with application of 50 μM staurosporine. As 20 μM glutamate is in the pathophysiological range for extracellular glutamate concentration in human traumatic brain injury patients [5], MK801 was assayed against this concentration.

*In vitro* studies on rodent neurons demonstrated the efficacy of NMDAR antagonists in preventing excitotoxic neuronal death [6]. To investigate the role of NMDARs in glutamate excitotoxicity in our system, enriched HESC-derived neuronal cultures were exposed to 20 μM glutamate in the presence and absence of MK801 (10 μM) for 24 h. MK801 conferred a significant neuroprotective effect against glutamate-induced excitotoxic injury (33.2 ± 8.4% cell death compared to control: 57.5 ± 3.4%; *p* < 0.05, Fig. 2d). When challenged with 150 μM NMDA, neuronal death was also observed (27.7 ± 3.6%; *p* < 0.001, Fig. 2e), however at lower levels than those induced by glutamate. Further work is required to elucidate the downstream signalling pathways that mediate glutamate and NMDA-induced excitotoxic neuronal dysfunction and death. Establishment of a robust platform for functional glutamate transmission and excitotoxicity in human neurons permits examination of these downstream pathways and could potentially aid the discovery of novel therapeutic targets.

## 4 Discussion

Pathological stimulation of NMDAR by glutamate has been implicated in excitotoxic injury and channel blockers have been trialled in human conditions to poor effect [20], suggesting that our understanding of human NMDAR-signalling is incomplete and reliable human-based experimental models could be informative, alongside animal studies. However, the development of glutamate-dependent currents was previously uncharacterised in enriched HESC-derived neurons *in vitro*.

In these studies, we demonstrate that HESC-derived neurons, within the context of the current experimental platform, acquired maturation-dependent glutamate-responsiveness over time in culture; knowledge of these characteristics is critical for the use of HESC-neural derivatives in models of neurological injury. By mRNA expression studies, there were large increases in transcription of *GRIN1* encoding the constitutive NMDAR1 subunit, *GRIA1* encoding the AMPAR1 subunit, and modest increases in further NMDAR and AMPAR subunit genes. These modest transcriptional increases translated to the expression of functional and properly assembled NMDARs as determined by functional studies herein, utilising NMDA as a specific agonist and the selective inhibitor D-APV. Furthermore, quantitative studies of current amplitude and the proportion of glutamate-responsive cells demonstrated gradual increase of receptor function over duration in culture, thus suggesting neuronal-intrinsic modulation of glutamate-receptor expression and functional downstream-signalling over time. Direct confirmation of NMDAR and AMPAR subunit profiles could be elucidated further by electrophysiological studies with appropriate subunit-specific inhibitors. The mRNA expression data also suggested that NMDAR subunit expression was dynamic over the *in vitro* time course, with GluN2B predominance. It remained to be seen whether the subunit profile would develop over time to recapitulate the conversion from GluN2B to GluN2A predominance in experimental mammalian systems [41].

In this study, glutamate was investigated as a physiologically relevant stressor due to its direct pathophysiological role in disease states. Dose-survival curves demonstrated that HESC-derived neurons underwent glutamate-induced cell death over the range of concentrations seen in human brain injury patients *in vivo* [5], and could be rescued with MK801 co-administration. Similar concentrations of glutamate and MK801 have also been demonstrated to mediate excitotoxic cell death and protection in rodent *in vitro* studies [7,37], and in human neurons derived from the human NT2 teratocarcinoma cell-line [14,32].

Interestingly, MK801 did not completely block glutamate-mediated neuronal death and, furthermore, NMDA alone induced a lower level of neuronal death than that mediated by glutamate. These findings suggest other mechanisms of glutamate-induced neuronal death in addition to NMDAR-mediated excitotoxicity. Glutamate in high concentrations has been demonstrated to cause oxidative-stress mediated neuronal death by competitively inhibiting cystine uptake *via* the Xct cystine/glutamate antiporter and impairing glutathione production [33,37]. While the concentrations used to experimentally induce glutamate-induced oxidative cell death are typically in the millimolar range [36], excitotoxic neuronal death at lower glutamate concentrations, mediated by NMDAR activation, has been shown to result in further glutamate release and accumulation in medium to higher concentrations capable of mediating oxidative neuronal death [37]. Calcium-permeable AMPARs, lacking the GluR2 subunit that prevents calcium permeability, have also been demonstrated to mediate hippocampal neuronal death in rodent slice cultures. Oxygen-glucose-deprivation increases synaptic-trafficking of GluR2-lacking AMPARs in CA1 hippocampal neurons, causing AMPAR mediated excitotoxic cell death [9]. The pre-exposure of HESC-neurons to trophically-deprived medium in the current experimental model may have affected the trafficking of AMPAR and partially accounted for the MK801-insensitive excitotoxic neuronal death observed.

## 5 Conclusions

Human pluripotent stem cell technology, including HESCs and iPSCs, has tremendous potential for modelling neurological disease processes. Technological advances have allowed the generation of enriched human neurons and glia under defined conditions, permitting study of human neural cells *in vitro* [13,24,35]. Here, we characterised a population of enriched HESC-derived neurons in terms of development of glutamatergic responses and investigated their utility in modelling glutamate-induced neuronal injury. We established that after 2 months in culture, HESC-derived neurons underwent excitotoxic cell death at concentrations of glutamate found in the brains of human patients suffering acquired brain injury, and thus have the potential to recapitulate aspects of human pathophysiology. Furthermore, insights from the development and refinement of HESC-based platforms for *in vitro* modelling of neuronal injury and rescue will be critical for the development of other HPSC-based complementary systems, including iPSCs.

## Conflict of interest

The authors declare no conflicts of interest and have approved the final article.
